# Understanding Determinants of Pregnant Women’s Knowledge of Lifestyle-Related Risk Factors: A Cross-Sectional Study

**DOI:** 10.3390/ijerph19020658

**Published:** 2022-01-07

**Authors:** Farah Nawabi, Franziska Krebs, Laura Lorenz, Arim Shukri, Adrienne Alayli, Stephanie Stock

**Affiliations:** Institute for Health Economics and Clinical Epidemiology (IGKE), Faculty of Medicine and University Hospital Cologne, University of Cologne, 50935 Cologne, Germany; franziska.krebs@uk-koeln.de (F.K.); laura.lorenz@uk-koeln.de (L.L.); arim.shukri@uk-koeln.de (A.S.); adrienne.alayli@uk-koeln.de (A.A.); stephanie.stock@uk-koeln.de (S.S.)

**Keywords:** questionnaire, knowledge, pregnancy, lifestyle, gestational weight gain, nutrition, physical activity, substance use

## Abstract

Research indicates that a woman’s lifestyle during pregnancy influences her child’s health and development. Therefore, women need to possess sufficient knowledge regarding the elements of a healthy lifestyle during pregnancy. To date, there has been little research on the assessment of lifestyle knowledge of pregnant women in the perinatal healthcare setting. This study describes the development and application of a knowledge-based questionnaire for pregnancy to be used in a lifestyle intervention trial conducted in Germany. Within the trial, pregnant women receive counselling on lifestyle topics. These topics are based on the German initiative ‘Healthy Start—Young Family Network’ (GiL), which provides evidence-based recommendations regarding diet and lifestyle before and during pregnancy. These serve as a basis for health professionals who provide counselling on healthy lifestyle choices during the antenatal period. The questionnaire consists of eight items, each of which can be answered using ‘Yes’, ‘No’ or ‘Don’t know’. The pregnant women who completed the questionnaire at baseline around the twelfth week of gestation were recruited within the host trial from gynaecological practices in Germany. Demographic variables and the respondents’ answers to the questionnaire were analysed using descriptive statistics and regression analyses. Descriptive statistics show that more than 85% of participants answered the majority of questions (n = 5) correctly. Questions on whether tap water is safe and the normal range for gestational weight gain (GWG) were answered correctly by about 62% and 74% of the women, respectively, and the question on whether it is beneficial to obtain information on breastfeeding at an early stage was answered correctly by about 29%. The results of the regression analyses indicate that age, gestational week, education and income are positive predictors for answering the questionnaire correctly. Nullipara and migration background are predictors for answering the questions incorrectly. This study indicates that there are gaps in women’s knowledge regarding lifestyle during pregnancy. Particular focus on certain topics, such as breastfeeding and normal GWG ranges, is still required during counselling. Our analysis shows that migration background is a predictor of insufficient knowledge and incorrect answers to the questions. Women with such backgrounds require special attention during antenatal counselling in order to cater to their needs and the gaps in their knowledge.

## 1. Introduction

Research indicates that a mother’s health behaviour during pregnancy influences her child’s health and development. Through a process referred to as perinatal programming, external factors, such as a pregnant woman’s lifestyle influence risks during pregnancy, birth complications and the child’s susceptibility to health impairments, such as obesity and chronic diseases [1,2,3]. The way the pregnant woman’s weight changes play a key role in this regard. Excessive weight gain increases the risk of birth complications and gestational diabetes [4,5,6], macrosomia [7], large for gestational age (LGA) [8] and obesity later in the child’s life [9]. Beneficial behaviours include exercise and physical activity, as these are positively associated with pregnancy outcomes, such as the reduction in LGA and gestational diabetes mellitus (GDM) [10], lower likelihood of preterm birth [11] and normal mode of delivery [12]. Alcohol consumption during pregnancy is a further risk factor and bears an increased risk of a variety of negative health outcomes for the offspring. These include growth defects, tissue and nerve damage and behavioural impairments [13]. The same holds true for smoking, which is also associated with negative health effects for the foetus, such as preterm birth, obesity and intellectual impairments [14].

Existing within the German antenatal healthcare setting, the ‘Healthy Start—Young Family Network’ (GiL) is an alliance that provides evidence-based recommendations for counselling on healthy lifestyle choices during pregnancy to health professionals involved during the antenatal period (e.g., gynaecologists, midwives, paediatricians) [15]. The network was established by the German Federal Centre for Nutrition (BZfE) and consists of a multidisciplinary scientific task force in the antenatal field. The information it provides on healthy lifestyle choices is tailored to specific target groups: either the aforementioned health professionals or families and women in the antenatal phase. The recommendations provided by the GiL are based on extensive systematic reviews, which were first published in 2012 and later updated in 2018 [15]. Regarding weight gain, the GiL recommends a range of 10–16 kg for women of normal weight, while about 10 kg is considered sufficient for overweight and obese women [15]. They also suggest for pregnant women to be physically active for at least 30 min, five days a week [15]. Guidelines on energy intake are difficult to find. However, a well-balanced diet, fruit, vegetables and wholegrain product consumption are recommended [16,17,18]. According to the German recommendations, energy requirements during pregnancy only increase by 10% in the last trimester [15]. The consumption of alcohol is advised against at both the national and the international level, as there is no available evidence regarding the amount of alcohol that can be consumed safely during pregnancy. Even if a pregnant woman does not smoke herself, passive smoking also bears risks, which is why pregnant women are additionally advised to avoid being in rooms where people are smoking [15,16,17,18]. Breastfeeding after birth is highly recommended wherever possible due to the benefits it offers both mother and child [19,20]. In order to enable them to follow a healthy lifestyle during pregnancy, it is necessary to ensure that women possess a full understanding of these health facts regarding the lifestyle factors that influence their child’s development during pregnancy. Since these recommendations are supposed to be communicated by healthcare providers during antenatal appointments, pregnant women should possess sufficient knowledge on these topics. However, research indicates that this is not the case: international studies show that women lack knowledge when it comes to pregnancy-related risk factors that might be harmful to the health and development of their unborn children, such as alcohol use, (passive) smoking, nutrition [21] and obesity in the mother [22,23].

Few existing questionnaires assess lifestyle knowledge among pregnant women. While questionnaires do exist on the assessment of separate topics, such as nutrition [24,25], nutrition and physical activity [26], nutrition and supplement intake [27], pregnancy-related risk factors [28] and alcohol consumption [29], we were unable to find a comprehensive questionnaire that assessed knowledge levels regarding both lifestyle and breastfeeding during pregnancy. Only one recent study conducted in Germany had developed a questionnaire covering lifestyle and expanded it to include topics such as dental health [30]. With its 22 items and multiple-choice and multiple-select answers, this tool might be impractical for a clinical setting. As such, we have developed a short, knowledge-based questionnaire on lifestyle during pregnancy in the antenatal healthcare setting that could be used as a screening instrument and provided initial results on its usage. The aim of this study is to evaluate the level of knowledge concerning lifestyle behaviour among pregnant women and the association between socio-demographic and pregnancy variables and knowledge levels.

## 2. Materials and Methods

For this cross-sectional study, we developed a knowledge-based questionnaire to be filled in by pregnant women around their twelfth week of gestation, which is at baseline, in the perinatal health service setting. The questionnaire was developed as part of the GeMuKi (acronym for ‘Gemeinsam Gesund: Vorsorge plus für Mutter und Kind’—Strengthening health promotion: enhanced check-up visits for mother and child) project, the host trial, which provides counselling on lifestyle topics in addition to regular antenatal care. Details on the project and its design can be found elsewhere [31,32]. According to the definition of health literacy provided by Sorensen et al. (2012), knowledge is a contributing factor to health literacy [33]. As such, we developed a knowledge questionnaire based on the topics that are communicated during counselling by a healthcare provider, which again are based on the GiL’s recommendations. The questionnaire was developed by the research team and was then discussed with subject matter experts from the study group. It was pre-tested on pregnant women (n = 8) at the Women’s Clinic at the University Hospital Cologne. Women were asked prior to their antenatal appointment whether they were willing to fill in the questionnaire as part of a questionnaire pre-test. In accordance with the principles of cognitive questionnaire pre-testing [34], the women were asked whether they had any remarks on the questionnaire after they had filled it in, particularly with regards to comprehension and the phrasing of the questions. Changes were made accordingly, and the questionnaire was finalised.

### 2.1. Description of the Knowledge Questionnaire

The questionnaire development process resulted in eight questions in total, which can be answered on a ‘Yes/No/Don’t know’ scale [31]. Table 1 provides an overview of the items that make up the questionnaire.

### 2.2. Sample

The study uses the sample and baseline data from the GeMuKi lifestyle counselling trial conducted in routine antenatal care settings in the state of Baden-Württemberg, Germany [32]. Gynaecologists participating in said trial asked eligible women if they were interested in taking part in the project. These women were handed a paper-based questionnaire at enrolment as a means of obtaining demographic data. After enrolment, they were asked to fill in the knowledge-based questionnaire using an app developed by the Fraunhofer Institute for Open Communication Systems (FOKUS), Berlin, Germany, for the purpose of the trial. Data collection took place from February 2019 to September 2021. They were analysed in November 2021 and entail a sample of 1466 women. The inclusion criteria for the host trial were as follows: on statutory health insurance (in Germany, health insurance is required by law and approximately 86% of the population are enrolled in the statutory insurance [35]), a patient at a participating gynaecological practice, signed informed consent, aged ≥18, proficient in German and not yet at the end of the twelfth week of gestation. Additionally, women with mental health issues were excluded, as they would be receiving specialised care.

### 2.3. Statistical Analysis

The questionnaire was statistically analysed using descriptive and inferential statistics such as frequency count, percentage and multiple regressions. In order to calculate the frequency with which questions were answered correctly, each correctly answered question was coded 1; incorrectly answered questions were coded 0. A sum score was built to display the overall score of the questionnaire by adding together the number of correct answers for every single question. The sum score thus ranged from 0 to 8. In order to allow for the application of regression models, the answer ‘Don’t know’ was coded as a missing value. In order to answer the question on the association between sociodemographic and pregnancy variables and knowledge levels, regression analyses were conducted. The women’s knowledge regarding lifestyle choices during pregnancy was subsequently selected as a dependent variable, and the individual questions and the sum score were used to build multiple logistic and linear regression models. The linear regression model used list-wise deletion. Age, nullipara, net income, migration background and educational level were used as independent variables. Age was used as a continuous variable. Nullipara and migration background were used as dichotomous variables (Yes/No). For migration background, an indicator was calculated using the following items: a person who was not born in Germany, whose parents were not born in Germany, who has moved to Germany at a later point in life or whose mother tongue is not German. Net income was categorised into percentiles and used as an ordinal variable. Educational level was categorised using the International Standard Classification of Education (ISCED) [36] and likewise used as an ordinal variable. For the purpose of this study, we adapted the following categorisation according to ISCED: primary, lower secondary, upper secondary, post-secondary-non-tertiary and university degree. The sample size was calculated for the initial trial using a different primary outcome than this study [32]. Marital status was excluded from the regression analysis since this variable was collected very vaguely by categorising it into single, married and divorced. A *p*-value of <0.05 indicated statistical significance. Tables with results of the regression analysis entail the total number of included cases. All the analyses were conducted using IBM^®^ SPSS^®^ Statistics for Windows, Version 28.0.

## 3. Results

Table 2 provides an overview of the demographic characteristics of the participants.

The mean age of the women who participated in the study was 33; half of the study population did not have any children at the time of participation (50.0%). The household net income was EUR 4295 per month. More than half of the participants had a university degree (55.1%) and were married (67.8%). Women with migration backgrounds represented 22.7% of the total sample.

Table 3 displays the results of the knowledge-based questions. The results indicate that most of the questions (n = 5) were answered correctly by the majority of women (more than 85% of participants). Questions on water intake and GWG were answered correctly by about 62% and 74% of the women, respectively, and the question on breastfeeding was answered correctly by about 29%.

None of the women scored one or zero points (Table 4). The majority of the women scored six or seven points (27.4% and 39.6%, respectively), and 16.8% percent scored eight points in the sum score.

### Variables Affecting Knowledge of Lifestyle-Related Risk Factors

Table 5 shows the linear regression model with the knowledge sum score as a dependent variable. Age, gestational week, education and income were positive predictors for the sum score, indicating that women with increased age, higher education levels, income and later gestational weeks possess significantly more knowledge regarding lifestyle factors during pregnancy. Nullipara and migration, on the other hand, were negative predictors. Note: while the residuals were normally distributed (histogram and p–p plot not shown), homoscedasticity was not evident (significant Breusch–Pagan test). Bootstrapping was performed to account for this uncertainty and confirmed the significance of the predictors of the regression model.

The logistic regression (Table 6) with the single questions indicates that there was a significant positive association between age and nullipara and the ability to answer the question on GWG correctly (OR = 1.067, 95% CI (1.000–1.139) and OR = 2.549, 95% CI (1.452–4.474), respectively). However, there was a significant negative association between nullipara and the question of whether or not it is safe to use tap water (OR = 0.375, 95% CI (0.262–0.537)). This differs for income, indicating that increased income was a predictor for the ability to answer the questions on tap water (OR = 1.080, 95% CI (1.009–1.155)), wholegrain products (OR = 1.254, 95% CI (1.118–1.406)), alcohol consumption (OR = 1.104, 95% CI (1.011–1.205)) and GWG (OR = 1.114, 95% CI (1.013–1.226)) correctly.

There was a significant negative association between migration background and the ability to answer the questions on alcohol consumption (OR = 0.454, 95% CI (0.288–0.714)), smoking (OR = 0.187, 95% CI (0.041–0.854)) physical activity (OR = 0.260, 95% CI (0.074–0.911)) and tap water (OR = 0.375, 95% CI (0.262–0.537)) correctly, indicating that women with migration backgrounds tended to answer these questions incorrectly. At the same time, migration was a positive predictor for answering the question on breastfeeding correctly (OR = 1.602, 95% CI (1.136–2.257)).

## 4. Discussion

The questionnaire described above assesses lifestyle knowledge among pregnant women in the German perinatal healthcare setting. The questionnaire particularly focuses on lifestyle during pregnancy, including GWG, nutrition, alcohol consumption, smoking, physical activity, water consumption and breastfeeding. Assessing lifestyle knowledge during pregnancy is essential, as research suggests that pregnant women lack knowledge when it comes to pregnancy-related information.

More than 83.8% of all the women surveyed produced a total score of six or more in the questionnaire. This is not surprising in light of the high level of education amongst our study population. However, it is surprising that the majority of the participants have a university degree, considering that people insured by all statutory health insurance funds took part in the study, including people with a wide range of socioeconomic statuses, and that the sample size is large enough to offer diversity. The highly educated population might also be a result of selection bias, as educated women possibly want to receive counselling on lifestyle topics because they are more interested as opposed to women with lower educational levels. Fewer women answered the question on the effectiveness of obtaining information on breastfeeding early during pregnancy correctly (36.6%) or did not know that this is beneficial (27.8%). This is a crucial result suggesting that pregnant women might not be aware that breastfeeding can come with difficulties. Preparing women for breastfeeding early by means of counselling might contribute to better-informed future mothers who have more realistic expectations about breastfeeding and seek help earlier. In Germany, the ‘Becoming Breastfeeding Friendly’ research project has developed recommendations to promote an environment that supports breastfeeding [37]. The effectiveness of such strategies is yet to be investigated. This is particularly important, as research suggests that engaging with the topic of breastfeeding early on during pregnancy supports the initiation and continuation of this approach [38].

As in the study by Oechsle et al. [30], the majority of our study population answered the questions on alcohol consumption and smoking correctly (85.7% and 90%, respectively). Moreover, the majority answered the questions on physical activity, portion size and wholegrain products correctly (90.5%, 93.2% and 87.2%, respectively). This could be attributed to the informative counselling that women may receive during their pregnancy and the fact that more than half of our study population possessed above-average educational levels (university degree). A study comparing all of Germany’s 16 federal states indicated that the state of Baden-Württemberg, from which the women in this study were retrieved, is ranked fourth in terms of education [39]. Nevertheless, knowledge gaps and different predictors for the correct and wrong answering of the question were identified.

Even though the majority of our study population answered the question on GWG correctly, a proportion of women do not know the normal range for weight gain during pregnancy. This finding suggests that a lack of knowledge on healthy weight gain may contribute to high rates of GWG above the normal range. It has implications for lifestyle counselling, which should address knowledge in addition to other influencing factors, such as social norms regarding the acceptability of weight gain during pregnancy that have been identified previously [40,41].

Particular focus must also be placed on the initial BMI of the women before or at the beginning of the pregnancy in order to ensure that they receive appropriate counselling on GWG [15]. The question on GWG was more likely to be answered by women who had no children at the time of participation. This might be because first-time mothers are more aware of and receptive to health information, as they want to be well-prepared for their first child. It is also possible that counselling on GWG has improved over time to the benefit of new mothers.

A migration background was a predictor for the questions on alcohol, smoking, physical activity and water consumption. In all cases, women with migration backgrounds were significantly more likely to answer these questions incorrectly. Women with a migration background might not know that alcohol consumption is harmful to their unborn child; however, research suggests that pregnant women with migration backgrounds are less likely to consume alcohol [42]. This might be explained by health beliefs and practices from their home countries, which they continue to follow even after migration [43,44]. An Israeli study of 3815 pregnant women on the awareness of alcohol consumption recommendations during pregnancy showed that no Muslim women reported alcohol consumption during pregnancy [45]. An analysis of this subgroup was not possible since we did not collect data on religious beliefs. The countries from which the parents of our sample migrated vary, covering Europe and beyond, from Turkey, Russia, Romania, Poland, Kazakhstan, to Italy, which is too widespread to make conclusions on health behaviour or possible religions and beliefs. With regards to the results and existing literature, a distinction has to be made between knowing a fact and behaving accordingly. Similarly, research on smoking indicates that in practice, a migration background in pregnant women is associated with less exposure to smoking [46]. Increased smoking behaviour only comes with higher acculturation, as indicated by a German study of pregnant women with Turkish backgrounds [47]. A migration background is a predictor for answering the breastfeeding question correctly. Studies indicate that immigration is associated with increased breastfeeding initiation [48,49]. This might be explained by cultural beliefs and traditions from the person’s country of origin and also by the possibility of immigrants living in communities that have closer ties to their homelands and traditions/cultures there that support breastfeeding [48].

According to our results, a substantial portion of pregnant women does not think that it is safe to drink tap water or do not know whether this is the case. In the context of Germany, however, it is possible to drink tap water and prepare milk or meals for children using tap water. The results of this study indicate that a migration background and nullipara are negative predictors for answering this question correctly, which might be due to tap water safety in the participants’ countries of origin. Participants with later gestation weeks and higher levels of education and income answered this item correctly. Research on the consumption of tap water in pregnant women in different countries is lacking, and further investigation is thus required.

One limitation of this study is the low complexity of the questionnaire, which might have not been discriminating enough. The questionnaire was developed so that all the women who participated in the study could answer the questionnaire, regardless of their educational levels. Therefore, we cannot preclude the possibility that the questions and answering scale might have been leading. Additionally, the results of this study indicate that the majority of women have a university degree. Moreover, the sample might not be representative. The inclusion of participants insured by all the statutory health insurance funds allowed for the inclusion of clientele with different socio-economic backgrounds. However, it appears that even with the inclusion of all statutory health insurance funds, our sample might not be representative, as it is outstandingly well-educated. This may be attributable to the gynaecologists who recruited the patients, who may have excluded potentially eligible pregnant women due to their socio-economic status or language barriers. In addition to that, women who are more interested in lifestyle topics and counselling might be higher educated and more likely to participate in the GeMuKi project [50]. Additionally, it is possible that women in the study regions simply are outstandingly well-educated, as the ranking within Germany showed.

## 5. Conclusions

This study indicates that women’s knowledge of lifestyle-related factors during pregnancy differs with regard to particular topics and socio-economic factors. Particular focus on certain topics, such as the benefits of early familiarisation with breastfeeding, the safety of tap water in Germany and the normal ranges of GWG with regards to the initial BMI of the woman, is required during antenatal counselling. Our analysis has shown that a migration background is a predictor for answering the questions on alcohol and smoking incorrectly. However, it is likely that pregnant women with a migration background still exhibit correct behaviour due to cultural beliefs retained from their homelands, such as not drinking alcohol as a general habit. Nevertheless, this group of women requires special attention during antenatal counselling in order to cover their needs and knowledge gaps.

## Figures and Tables

**Table 1:** Knowledge questionnaire.

Topic	Question
GWG	Is it generally recommended for women of normal weight to gain 20 kg during pregnancy?
Portion size	Do pregnant women have to eat larger portions right from the start of their pregnancy to make sure that the baby gets enough food?
Alcohol	Can even small amounts of alcohol harm the unborn baby at any point during pregnancy?
Smoking	Does it harm the unborn child if people smoke around the pregnant woman (passive smoking)?
Physical activity	Does it harm the unborn child if women exercise during pregnancy?
Breastfeeding	Does breastfeeding work better the earlier a pregnant woman receives information about breastfeeding?
Water	Is tap water just as good for a pregnant woman as bottled mineral water?
Whole grains	Are wholegrain products usually the better choice if you want to eat pasta, bread or rice while pregnant?

**Table 2:** Sample characteristics.

Characteristics	
Age	32.8 years (SD 4.37)
Nullipara	50.0% (n = 711/1422)
Migrant	22.7% (n = 329/1447)
Income	Mean: EUR 4295
	Percentile
	25 = EUR 3250
	50 = EUR 4250
	75 = EUR 5200
Education level	
Primary	0.1% (n = 2/1404)
Lower secondary	2.8% (n = 39/1404)
Upper secondary	9.9% (n = 139/1404)
Post-secondary-non-tertiary	32.1% (n = 451/1404)
University degree	55.1% (n = 773/1404)
Marital status	
Single	30.1% (n = 425/1412)
Married	67.8% (n = 958/1412)
Divorced	2.1% (n = 29/1412)

Note: percentages are provided for categorical variables and means for continuous variables. EUR = Euro.

**Table 3:** Evaluation of questionnaire.

Topic	Correct Answer	Incorrect Answer	Do Not Know	Missing
GWG	1083 (73.9)	76 (5.2)	208 (14.2)	99 (6.8)
Portion size	1366 (93.2)	5 (0.3)	6 (0.4)	89 (6.1)
Alcohol	1257 (85.7)	109 (7.4)	12 (0.8)	88 (6.0)
Smoking	1320 (90)	8 (0.5)	46 (3.1)	92 (6.3)
Physical activity	1327 (90.5)	13 (0.9)	36 (2.5)	90 (6.1)
Breastfeeding	426 (29.1)	541 (36.9)	408 (27.8)	91 (6.2)
Water	909 (62)	247 (16.8)	215 (14.7)	95 (6.5)
Whole grains	1279 (87.2)	41 (2.8)	54 (3.7)	92 (6.3)

Note: results are displayed as n (%).

**Table 4:** Sum score of knowledge-based questionnaire (missing n = 88 (6%)).

Score	N	%
2	3	0.3
3	11	1.1
4	40	3.2
5	146	11.7
6	345	27.4
7	485	39.6
8	211	16.8

**Table 5:** Linear regression model (dependent variable = sum score, n = 1191).

Independent Variables	R^2^	B	SE	*p*	95% CI
Model fit	0.116				
Age		0.016	0.008	0.040 *	0.001–0.031
Gestation week		0.037	0.015	0.017 *	0.007–0.067
Nullipara		−0.248	0.064	0.000 ***	−0.373–−0.122
Education level		0.142	0.027	0.000 ***	0.090–0.194
Migrantion background		−0.377	0.073	0.000 ***	−0.520–−0.235
Income		0.065	0.013	0.000 ***	0.040–0.090

* *p* < 0.05; *** *p* < 0.001.

**Table 6:** Logistic regression with single questions.

Independent Variables			Age			Gestation Week			Nullipara			Education			Migration Background			Income		
Dependent Variable	R^2^_Nagel-kerke_	*p*_Hosmer-Lemeshow_	OR	95% CI	*p*	OR	95% CI	*p*	OR	95% CI	*p*	OR	95% CI	*p*	OR	95% CI	*p*	OR	95% CI	*p*
**GWG** **(n = 1010)**	0.073	0.958	1.067	1.000–1.139	0.050	1.058	0.931–1.203	0.384	2.549	1.452–4.474	0.001	1.079	0.875–1.331	0.478	1.052	0.575–1.923	0.870	1.114	1.013–1.226	0.027
**Portion size** **(n = 1188)**	0.157	0.439	1.105	0.853–1.432	0.449	1.032	0.600–1.775	0.910	0.000	0.000	0.992	1.167	0.473–2.882	0.737	0.841	0.085–8.270	0.882	1.143	0.812–1.608	0.444
**Alcohol** **(n = 1183)**	0.037	0.799	0.968	0.916–1.022	0.240	1.008	0.901–1.128	0.890	0.748	0.471–1.188	0.219	1.028	0.851–1.242	0.775	0.454	0.288–0.714	0.000	1.106	1.017–1.204	0.019
**Smoking** **(n = 1152)**	0.116	0.915	1.143	0.920–1.422	0.228	1.252	0.861–1.820	0.240	1.516	0.288–7.992	0.624	0.640	0.311–1.315	0.225	0.187	0.041–0.854	0.030	1.207	0.904–1.610	0.202
**Physical activity** **(n = 1161)**	0.063	0.909	0.982	0.839–1.149	0.818	0.914	0.655–1.276	0.597	0.511	0.130–2.000	0.335	0.911	0.516–1.608	0.747	0.260	0.074–0.911	0.035	1.167	0.933–1.460	0.176
**Breastfeeding** **(n = 850)**	0.022	0.235	1.024	0.987–1.063	0.200	1.017	0.946–1.094	0.641	1.165	0.862–1.573	0.320	1.119	0.990–1.265	0.072	1.602	1.136–2.257	0.007	0.982	0.925–1.043	0.562
**Water** **(n = 1012)**	0.125	0.568	1.004	0.963–1.046	0.868	1.158	1.063–1.262	0.000	0.465	0.327–0.663	0.000	1.346	1.168–1.550	0.000	0.375	0.262–0.537	0.000	1.080	1.009–1.155	0.026
**Whole Grains** **(n = 1142)**	0.081	0.921	1.027	0.943–1.119	0.541	1.006	0.839–1.207	0.947	0.783	0.376–1.628	0.512	1.020	0.760–1.370	0.895	0.523	0.253–1.079	0.079	1.254	1.118–1.406	0.000

There was a significant positive association between gestational week and education and the question on tap water consumption, indicating that women with later gestational weeks (OR = 1.158, 95% CI (1.063–1.262)) and higher education levels (OR = 1.346, 95% CI (1.168–1.550)) tended to answer this question correctly.

## Data Availability

The datasets used and/or analysed during this study are available from the corresponding author on reasonable request.
